# Comparative evaluation of fluorescent in situ hybridization and Giemsa microscopy with quantitative real-time PCR technique in detecting malaria parasites in a holoendemic region of Kenya

**DOI:** 10.1186/s12936-017-1943-4

**Published:** 2017-07-24

**Authors:** Joseph Osoga, John Waitumbi, Bernard Guyah, James Sande, Cornel Arima, Michael Ayaya, Caroline Moseti, Collins Morang’a, Martin Wahome, Rachel Achilla, George Awinda, Nancy Nyakoe, Elizabeth Wanja

**Affiliations:** 1 Malaria Diagnostics Center, Kenya Medical Research Institute/United States Army Medical Research Directorate-Kenya, Box 54, Kisumu, 40100 Kenya; 2 Basic Sciences Laboratory, Kenya Medical Research Institute/United States Army Medical Research Directorate-Kenya, Box 54, Kisumu, 40100 Kenya; 3 Biomedical Sciences and Technology Department, School of Public Health and Community Development, Maseno University, Box Private Bag, Maseno, Kenya; 4 United States Army Medical Research Directorate-Armed Forces Research Institute of Medical Sciences, Bangkok, 10400 Thailand

**Keywords:** Malaria, P-Genus fluorescent in situ hybridization, Microscopy, Quantitative real-time PCR, Performance

## Abstract

**Background:**

Early and accurate diagnosis of malaria is important in treatment as well as in the clinical evaluation of drugs and vaccines. Evaluation of Giemsa-stained smears remains the gold standard for malaria diagnosis, although diagnostic errors and potential bias estimates of protective efficacy have been reported in practice. *Plasmodium* genus fluorescent in situ hybridization (P-Genus FISH) is a microscopy-based method that uses fluorescent labelled oligonucleotide probes targeted to pathogen specific ribosomal RNA fragments to detect malaria parasites in whole blood. This study sought to evaluate the diagnostic performance of P-Genus FISH alongside Giemsa microscopy compared to quantitative reverse transcription polymerase chain reaction (qRT-PCR) in a clinical setting.

**Method:**

Five hundred study participants were recruited prospectively and screened for *Plasmodium* parasites by P-Genus FISH assay, and Giemsa microscopy. The microscopic methods were performed by two trained personnel and were blinded, and if the results were discordant a third reading was performed as a tie breaker. The diagnostic performance of both methods was evaluated against qRT-PCR as a more sensitive method.

**Results:**

The number of *Plasmodium* positive cases was 26.8% by P-Genus FISH, 33.2% by Giemsa microscopy, and 51.2% by qRT-PCR. The three methods had 46.8% concordant results with 61 positive cases and 173 negative cases. Compared to qRT-PCR the sensitivity and specificity of P-Genus FISH assay was 29.3 and 75.8%, respectively, while microscopy had 58.2 and 93.0% respectively. Microscopy had a higher positive and negative predictive values (89.8 and 68.0% respectively) compared to P-Genus FISH (56.0 and 50.5%). In overall, microscopy had a good measure of agreement (76%, k = 0.51) compared to P-Genus FISH (52%, k = 0.05).

**Conclusion:**

The diagnostic performance of P-Genus FISH was shown to be inferior to Giemsa microscopy in the clinical samples. This hinders the possible application of the method in the field despite the many advantages of the method especially diagnosis of low parasite density infections. The P-Genus assay has great potential but application of the method in clinical setting would rely on extensive training of microscopist and continuous proficiency testing.

## Background

Early and accurate diagnosis of malaria is critical in providing effective care and treatment as well as reducing morbidity and mortality. Accurate malaria diagnosis is also critical in evaluating anti-malarial drugs or vaccines [1]. Errors in malaria diagnosis (false positives, false negatives, and inaccurate species identification) may lead to biased estimates of protective efficacy. Microscopic diagnosis is considered to be the reference standard for determining the protective efficacy of prophylactic drugs or vaccines. However, microscopy has many inherent limitations [2], including the need for highly experienced technicians, variability in smear quality, inability to determine malaria species at low parasitaemia since a trained microscopist can realistically detect 50–100 parasites/µLof blood [3], artifacts resembling malaria, and loss of slide quality with time. The microscopy method is cumbersome and takes more than 30 min per slide in clinical diagnosis and between 1 and 2 h per slide in research setting [4].

The development of molecular techniques has provided new methods for identifying parasites nucleic acids. The challenges of microscopy have been overcome by polymerase chain reaction (PCR) which is a powerful and sensitive tool for malaria diagnosis capable of detecting very low parasite densities. However, its applications in clinical drug trials has been challenged by cases of false positive results since nucleic acids such as DNA remains in the blood long after the parasite has been cleared [5]. Moreover, the method is time-consuming and expensive and is therefore not generally used in initial malaria parasite detection and treatment of malaria patients. In the background of these challenges, there is a need to improve on microscopy to detect low parasitaemia and gametocytes concentrations. One such method is to label malaria parasites with fluorescent dyes and then use microscopy to visualize the labelled parasites.

Fluorescent in situ hybridization (FISH) technology has been used to detect infectious disease-causing agents in clinical samples [6]. FISH uses fluorescent labelled oligonucleotide probes targeted to pathogen specific ribosomal RNA fragments. A rapid, simple and specific FISH assay that can be used to diagnose malaria in low-resource malaria endemic countries has been developed [7]. There are various reasons why FISH is a promising technique in malaria diagnosis. First, it is based on rRNA which is highly abundant in the cell cytoplasm and therefore can be visualized using sequence-specific fluorescence probes under a microscope, without the need to perform target sequence amplification, and secondly, due to variable nucleotide sequence conservation, it is possible to find short nucleotide stretches that are unique to the genus, species, sub-species or strain [8].

This study sought to evaluate the performance of the P-Genus FISH research test kit (PlasGFK04 Palo Alto, California) and Giemsa microscopy against qRT-PCR (Applied Biosystems 7500 fast Real-Time PCR System, Life Technologies, USA) in detecting malaria parasites in patients with malaria-like symptoms in a holo-endemic area of western Kenya. Although PCR and FISH are all molecular based methods, FISH is a fluorescent microscopy-based technique that detects live parasites where rRNA is present in high copy numbers [9].

## Methods

### Study site

Study samples were collected between November 2013 and May 2014 at the Kenya Medical Research Institute (KEMRI)/Walter Reed Project (WRP) Clinical Trial Centre in Kombewa division of Kisumu West Sub-County, Kenya. This area is holoendemic for malaria with peak transmission occurring during the long rains (March–May) and short rains (November–February). Annual cumulative malaria attack rates in Kombewa (approximately 130,000 persons) for *Plasmodium falciparum* are about 95% during long rains and 75% during short rains [10].

### Study design and sample collection

This was a laboratory-based experimental study. Field samples were collected from 500 consenting volunteers with suspected malaria. Blood was collected through venepuncture by phlebotomists utilizing a blood draw standard operating procedure and universal precautions. Blood was obtained from veins on the ventral surface of the forearm or heel prick for infants. A total of 250 µL of whole blood was collected into an EDTA microtainer. All the samples were labelled with a study identification code which was unique to each study participant.

### Giemsa microscopy

Thick and thin malaria blood films (MBFs) were both prepared on the same slide. After air drying thick and thin films, only thin MBFs were fixed in absolute methanol. Both films were stained using 3% buffered Giemsa solution for 60 min. One blood film per sample was examined by two blinded independent expert microscopist at 1000× oil immersion fields. The thick film was examined and at least 100 high power fields were examined for a slide to be recorded negative. Parasite counting was performed by estimating the number of asexual parasites in 200 white blood cells (WBC) or 500 WBCs for very low density slides. Parasite density was estimated against the WHO recommended 8000 WBCs per microlitre of blood. Blinding was done to ensure quality of the microscopic examinations and in case there was a discordant reading between the two readers a third reading was performed by an expert microscopist. The expert microscopists were also involved in reporting and identifying the different species of parasites using fluorescent microscope and light microscope.

### Fluorescent in situ hybridization assay

Fluorescent in situ hybridization assay training was provided to expert microscopists from the Malaria Diagnostics Centre, Kisumu before commencement of the study. Training included thin smear preparation, conducting the assay procedure and microscopic identification of the *Plasmodium* parasites using a fluorescent microscope. *Plasmodium* genus FISH (P-Genus FISH) kit, (catalogue# PlasGK04) was provided by ID-FISH Technology Inc., Palo Alto, CA, USA. All assay kits contained relevant rRNA-specific probes, Smear Preparation Reagent (SPR), 2.5× *Plasmodium* Wash Buffer, 10× *Plasmodium* rinse buffer and *Plasmodium* counter-stain. The assays were performed according to the manufacturer’s instructions provided with the kits. The thin smears were observed under the fluorescent microscope green filter which has an excitation of 560 nm and an emission of 630 nm; the *Plasmodium* genus probe which was labelled with Texas Red dye appeared red during observation. The microscopic examination was performed by two blinded independent readers and examination was performed up to 300HPF (high power fields) before determining a slide was negative.

### Real-Time PCR

Total nucleic acid was extracted from frozen or whole blood using QIAamp DNA Blood Mini Kit (Qiagen, Valencia, CA) as recommended by the manufacturer. Molecular identification and quantification was performed by amplification of *Plasmodium* 18S rRNA on Applied Biosystems 7500 Fast real time PCR systems (Life technologies Grand Island, NY). For qRT-PCR, a reverse transcriptase step of 30 min at 50 °C incubation was included. This was followed a 10 min denaturation step, then 40 amplification cycles of 15 s at 95 °C denaturation and 1 min at 60 °C. The final reaction volume was 10 μL consisting of 1 μL of the template, 1× QuantiTect probe RT PCR master mix (Qiagen), 0.4 μM each primer, 0.2 μM probe and MgCl_2_ at a final concentration of 4 μM. Nucleic acid from cultured *P. falciparum* NF54 strain available in the laboratory was used as a positive control. Non template negative control contained nuclease free water (Life Technologies, Grand Island, NY).

### Primer design

Primer sets were based on *Plasmodium* 18S rRNA sequences deposited in the GenBank and designed using web-based software Primer3 v.0.4.0 (http://frodo.wi.mit.edu/primer3/). The primers were designed to amplify all units of rRNA distributed in all chromosomes: 1, 5, 7, 11 and 13. The regions of sequences selected were highly conserved and found only in the genus *Plasmodium.* The sequence of the forward primer was 5′-GCTCTTTCTTGATTTCTTGGATG-3′, and that of the reverse primer was 5′-ATGGCCGTTTTTAGTAAGATCTCGTTCG-3′ [11]. The probe sequence was 5′-ATGGCCGTTTTTAGTTCGTG-3′ labelled with 5′FAM (6-carboxyfluorescein) and 3′TAMRA (6-carboxytetramethyl-rhodamine) as the reporter and quencher sequences, respectively.

### Data analysis

Data was analysed using STATA version 13 for Windows. Frequencies and percentages were used to present the malaria results for FISH, Giemsa microscopy and qRT-PCR. Sensitivity, specificity, positive and negative predictive values (with 95% confidence intervals) of Giemsa microscopy and FISH were compared to qRT-PCR which was taken as a reference standard for malaria detection. Inter-test agreement for both results of positive and negative readings was expressed by the percentage of overall agreement and kappa statistic (κ) for the agreement between FISH, microscopy and RT-PCR.

## Results

### Comparison of P-Genus FISH, Giemsa microscopy and qRT-PCR for the detection of *Plasmodium* parasites in clinical samples

**Fig. 1:**
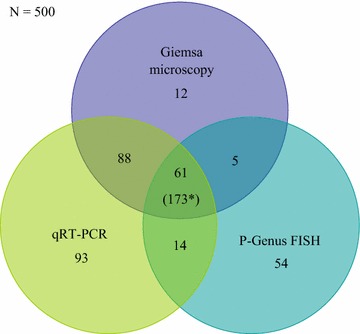
Venn diagram showing the relationship of malaria positive samples for all the three methods, (173*) negative cases

In this study, 500 participants with suspected malaria cases were recruited based on clinical presentation and screened for *Plasmodium* parasites by Giemsa microscopy, P-Genus FISH microscopy, and qRT-PCR. The study participants comprised majorly of children (58%, n = 290) less than 12 years and the rest were older children and adults (42%, n = 210) of 13−64 years of age. Majority of the participants were female (64%, n = 319); one woman was pregnant, 39% (n = 319) were not pregnant and the remaining were children. The numbers of *Plasmodium* positive cases were 26.8% (134/500) by P-Genus FISH, 33.2% (166/500) by Giemsa microscopy, and 51.2% (256/500) by qRT-PCR. The number of *Plasmodium* negative samples was 73.2% (366/500) by P-Genus FISH, 66.8% (334/500) by Giemsa microscopy, and 48.8% (244/500) by qRT-PCR. The relationship between P-Genus FISH, Giemsa microscopy, and qRT-PCR positive samples is highlighted in Fig. 1.

### Performance of P-genus FISH, Giemsa microscopy against qRT-PCR as the reference method

The P-Genus FISH assay had a low ability in detecting true malaria positive cases but a relatively higher ability to detect malaria negative cases. This is indicated by the sensitivity and specificity of P-Genus FISH (29.3 and 75.8% respectively). Microscopy showed a higher sensitivity (59.8%) and specificity (93%) than P-Genus FISH. Similarly microscopy had higher predictive values than P-Genus FISH in correctly determining malaria positive or negative cases. The positive and negative predictive values for P-Genus FISH were 56.0 and 50.5%, respectively, as compared to microscopy which had 89.8 and 68.0%, respectively. The positive likelihood ratios also indicate that microscopy performed better than P-Genus FISH (8.35 vs. 1.21), but it had a slightly better performance than microscopy in the negative likelihood ratios (0.43 vs. 0.93). This data indicate that microscopy performed better than P-Genus FISH in detection of *Plasmodium* parasites in 500 clinical samples. The estimated level of agreement between P-Genus FISH and qRT-PCR was lower (52%, k = 0.05) as compared to the level of agreement between microscopy and qRT-PCR (76%, k = 0.5), Table 1.

**Table 1:** Performance comparison of P-Genus FISH and Giemsa microscopy against qRT-PCR for the detection of malaria parasites in clinical samples

	Sensitivity % (95% CI)	Specificity % (95% CI)	Predictive value		Likelihood ratio		Methods agreement %	K-value
			Positive test % (95% CI)	Negative test % (95% CI)	Positive test % (95% CI)	Negative test % (95% CI)		
P-Genus FISH	29.3 (23.8–35.3)	75.8 (69.9–81.1)	56.0 (47.1–64.5)	50.5 (45.3–55.8)	1.21 (0.90–1.62)	0.93 (0.84–1.04)	52.0	0.051
Microscopy	58.2 (51.9–64.3)	93.0 (89.1–95.9)	89.8 (84.1–93.9)	68.0 (62.7–72.9)	8.35 (5.22–13.37)	0.45 (0.39–0.52)	75.2	0.508

*CI* confidence interval, *P-Genus FISH Plasmodium* genus fluorescent in situ hybridization, *K*-*value* kappa value

The extrapolated qRT-PCR parasite densities were stratified as ≤2 P/µL (n = 84), >2≤50 P/µL (n = 61), >50≤200 P/µL (n = 31), >200≤2000 P/µL (n = 28), and >2000 P/µL (n = 52) to test whether sensitivity of the methods was parasite density dependant. The P-Genus FISH showed a highly irregular pattern in detecting true malaria parasites at different PCR determined parasite densities. The P-Genus FISH had a high sensitivity (90.5%) at very low parasite density (<2 P/µL) compared to microscopy (38.1%), indicating the P-Genus FISH performs well at very low densities than microscopy. But as parasite density increases to 2–50 P/µL, surprisingly the sensitivity of P-Genus FISH declines significantly to 19.7%, but it gradually recovers with increased parasite density to 42% at >2000 P/µL. The sensitivity of microscopy increased with increase in parasite densities; from 38.1% at <2 P/µL to 92.3% at >2000 P/µL, Table 2.

**Table 2:** Sensitivity of P-Genus FISH and Giemsa microscopy compared to qRT-PCR classified by parasite densities

	≤2 P/µL (n = 84)	>2≤50 P/µL (n = 61)	>50≤200 P/µL (n = 31)	>200≤2000 P/µL (n = 28)	>2000 P/µL (n = 52)
P-Genus FISH (%)	90.48	19.67	25.81	57.14	42.31
Microscopy (%)	38.1	62.3	74.19	89.29	92.31

*P-Genus FISH Plasmodium* genus Fluorescent in situ hybridization, *µL* microlitre

## Discussion

Microscopic examination of Giemsa-stained thick blood films is still the most widespread method for the diagnosis of malaria [12]. The method is affordable, relatively fast, and allows species identification as well as quantification of parasites. But the method has several limitations such as false positive and negative results in non-experienced hands, errors in species identification, and incorrect parasite density estimations [13]. Some of the challenges of microscopy have been overcome by polymerase chain reaction (PCR) which is a powerful and sensitive tool for malaria diagnosis capable of detecting very low parasite densities. However, the applications of PCR in low resource endemic areas for clinical diagnosis have remained limited because accessibility is still restrictive, a time consuming technique and the diagnostic cost is high [14]. Fluorescent in situ hybridization (FISH) technology has been used to detect infectious disease-causing agents in clinical samples [6]. FISH uses fluorescent-labelled oligonucleotide probes targeted to pathogen specific ribosomal RNA fragments. It is a rapid, relatively inexpensive, and easy to perform test for the detection of *Plasmodium* parasites directly on a fixed thin smear [15].

The present study shows that the P-Genus FISH assay detected less malaria positive cases than microscopy, while qRT-PCR which was used as the reference method identified more positive cases than both methods. The PCR method can detect sub-microscopic parasites and has been shown to have a lower detection limit of 0.002–30 parasites/µL for monoplex assays while the multiplex assays have been shown to have a limit of 0.2–5 parasites/µL [16]. The present study highlights that the agreement level between the three methods was very low (Fig. 1) because only 61 positive and 173 negative cases were identified by the three methods. Furthermore the P-Genus FISH assay had the least number of shared positive cases with qRT-PCR, a feature that is highlighted by the low agreement kappa value (k = 0.05). Although both methods detect nucleic acids of the *Plasmodium* parasite, qRT-PCR is based on amplification and, therefore, likely to detect parasitaemia at very low threshold compared to P-Genus FISH assay that does not amplify. Therefore, differences in the detection methods of the two assays probably contributed to the low agreements [7, 11].

The present study indicates that microscopy had a higher diagnostic performance than P-Genus FISH assay. This can also be attributed to the differences between the detection methods. Both methods are microscopic but the P-Genus method identifies 18S-rRNA in live parasites while microscopy relies on detection of whole Giemsa-stained live or dead parasite [7, 13]. The expected limit of detection that can be achieved by an experienced microscopist for the examination of the thick blood film procedure is about 50 parasites/µL of blood [11, 17] while Shah et al. established that the limit of detection for FISH assay can be 1–2 parasites per 300 fields with a 100× objective microscope [7]. The current study indicates that sensitivity of microscopy was 58.2%, a value that is comparable to 50% obtained in a previous study that evaluated microscopy against PCR [18]. But the sensitivity of microscopy has been shown in a previous study that it can increase up-to 93% if demographics such as travel history, previous or past infections, and drug intake are considered [19].

Despite the fact that PCR was used, which is a more sensitive method for evaluating diagnostic performance, the sensitivity of the FISH assay was poor in this study as compared to a previous study by Shah et al. which showed that the sensitivity of the P-Genus FISH assay was 98% when compared to Giemsa microscopy [7]. The specificity of P-Genus FISH in the current study was 75.8% which is closely similar to the previous study (83.4%) by Shah et al. although she compared her study with Giemsa microscopy. The low sensitivity can be due to several challenges, including lack of a re-reading paradigm, lack of experienced microscopist to identify and quantify the *Plasmodium* species, presence of artifacts and other fluorescing objects.

The study was based in an endemic region where malaria prevalence is 27% [20], this makes the predictive values estimated in this study very important. The P-Genus FISH assay does not give any confidence to be used in clinical setting for the confirmation of positive or negative test patients. A positive predictive value of 56% indicates that the P-Genus FISH assay has ~50 rates of over-diagnosis which can result in high estimates of malaria morbidity and overuse of malaria drugs leading to treatment failures and drug resistance [21]. Similarly, the negative predictive value is also low (50%) which can increases the risk of missing detectable malaria parasites in infected individuals if the method is used in clinical diagnosis. Unlike P-Genus FISH, microscopy had a good positive predictive value (90%) with a range of up-to 94%, in essence it had less rates (>6%) of over diagnosis of malaria in the evaluated individuals, and this results supports its continued use as the conventional gold standard for malaria diagnosis [12].

Despite the inconsistencies observed in the stratified qRT-PCR parasite densities, the P-Genus assay shows a positive capability in identifying very low parasite densities. It had a sensitivity of 90% among 84 samples with <2 P/µL as compared to microscopy which had a sensitivity of 38.1% at the same density. The unique property of the P-Genus FISH assay to detect rRNA makes is almost equivalent to qRT-PCR in detecting low density parasites [7, 8, 15, 22]. But, as observed there are challenges in identification of the *Plasmodium* rRNA parasites in thin smears as parasitaemia increases and this could be attributed to high fluorescence of the films under the microscope (including artifacts) resulting in the incorrect judgment by the microscopist. It remains to be seen whether, with intensive proper training and field experience the assay can be applied in clinical diagnosis in resource poor setting, because among other advantages [7] it can detect low density parasites and provide morphological information of the infecting species.

## Conclusion

The P-Genus FISH assay is easy to perform if mastered properly and only requires an LED unit with special fluorescent filters attached onto a light microscope. However, the diagnostic performance of the method in clinical samples has been shown to be low when compared to qRT-PCR. The study also exposed the challenges facing microscopy especially in low parasite densities; but still it performed better than P-Genus FISH in detection of malaria parasites. The P-Genus FISH assay has great potential in detecting low density parasites. It remains to be seen whether, with intensive proper training and field experience the assay can be applied in clinical diagnosis. If not, the assay will need to be improved.
